# The presence of multiple gestational sacs confers a higher live birth rate in women with infertility who achieve a positive pregnancy test after fresh and frozen embryo transfer: a retrospective local cohort

**DOI:** 10.1186/1477-7827-12-104

**Published:** 2014-11-24

**Authors:** Erika Balassiano, Shaveta Malik, Praful Vaid, Eric S Knochenhauer, Michael L Traub

**Affiliations:** Staten Island University Hospital, 475 Seaview Avenue, Staten Island, NY 10305 USA; Island Reproductive Services, 1110 South Avenue Suite 305, Staten Island, NY 10314 USA

**Keywords:** In-vitro fertilization (IVF), Frozen embryo transfer (FET), Miscarriage, Multiple pregnancy

## Abstract

**Background:**

After spontaneous conception, the rate of miscarriage is more common in multiple rather than singleton pregnancies. However, the incidence of miscarriage is lower in in-vitro fertilization twin versus singleton pregnancies. Most patients have little understanding of pregnancy outcomes once they achieve a positive pregnancy test. This study investigated the relationship between multiple pregnancy and miscarriage in women with infertility after fresh and frozen embryo transfer.

**Methods:**

Retrospective local cohort study of all consecutive patients undergoing in-vitro fertilization at our institution (n = 1130), fresh or frozen embryo transfer, between January 1, 2008 and December 31, 2012. Patient characteristics (age, body mass index, initial hCG, maximum follicle stimulating hormone levels) and in-vitro fertilization parameters (estradiol levels, eggs retrieved, and endometrial thickness) were collected and statistically analyzed using *T*-test and Chi-square test (Stata version 10). Linear and logistic regression were used when appropriate.

**Results:**

Overall, live birth rate for all cycles was 30.44% and total pregnancy loss was 6.55% - similar for fresh and frozen cycles despite a higher rate of biochemical pregnancies for frozen cycles. Among all pregnant patients, 62.48% had a live birth. Although clinical pregnancy rate was higher for fresh cycles, live birth rates were similar. In pregnancies where multiple sacs were demonstrated on ultrasound, live birth rates were higher despite 31% of patients losing at least one sac. This finding was comparable between fresh and frozen cycles. However, in patients under age 35 and using donor egg, no live birth advantage was seen in patients with multiple sacs. In fact, transferring more than one embryo did not increase live birth rate either.

**Conclusions:**

Despite the many maternal and fetal risks of multiple pregnancies, patients who achieve a positive pregnancy test with fresh and frozen in-vitro fertilization and who have more than one pregnancy sac are more likely ultimately to deliver at least one baby. This finding is true of both fresh and frozen embryo transfer cycles. This pregnancy advantage is not seen in young patients and in patients using donor egg, and single embryo transfer maximizes birth outcomes.

## Background

Early pregnancy loss is unfortunately a common clinical event. Between four and twenty weeks gestational age, approximately 12-15% of clinically recognized pregnancies end in miscarriage. This number can be two to four fold greater if unrecognized early miscarriages are included. As many as 60% of all conceptions abort within the first trimester and at least 50% of all losses go unnoticed [1, 2].

In spontaneous conceptions, miscarriage is more common with multiple pregnancy. For many decades it has been suggested that twins are more often conceived than born [3]. More than thirty years later, other studies confirmed his hypothesis: three times more twins were identified among aborted pregnancies than term pregnancies [4, 5]. The true prevalence of multiple pregnancy sacs or multiple fetal heartbeats in early pregnancies is not known due to undercounting [6] and vanishing twins [7, 8].

However, in vitro fertilization (IVF) studies show lower rates of miscarriage with twin gestations than singletons [9–11]. This is true for total pregnancy loss (loss of all sacs and fetal heart beats) as well as for pregnancy loss per gestational sac in multiple pregnancies when compared to singletons [9]. Due to earlier and closer clinical follow up of artificial reproductive technology (ART) pregnancies, more multiple gestational sacs and heartbeats are likely recognized in these women than in spontaneous conceptions from fertile women.

Pregnancy loss is known to occur for embryonic and maternal factors, but many times no answer is found. Aneuploidy likely accounts for a significant amount [12]. Relatively little is understood about the rate of pregnancy loss among fresh and frozen embryos since miscarriages still occur in women with a normal uterine cavity and with known euploid embryos. This constrains physician counselling regarding the number of embryos to transfer. In addition to managing patient expectations with regards to achieving a positive pregnancy test, it is equally important to counsel patients on pregnancy outcomes once they achieve their initial positive pregnancy test.

In the current study, we aim to quantify the chance of live birth and intermediate pregnancy outcomes in women with an initial positive pregnancy test and determine if this outcome differs between fresh IVF and frozen embryo transfer (FET) cycles. Additionally, we investigate the relationship between multiple pregnancy and pregnancy loss in both fresh and frozen cycles.

## Methods

We performed a retrospective local cohort study of all consecutive patients undergoing IVF in a single, private center at Island Reproductive Services, Staten Island, NY, between January 1st, 2008 and December 31st, 2012. Medical records were reviewed for a total of 1130 ART cycles in which transfer took place with either IVF or FET cycle. Cycles were excluded if they did not result in embryo transfer (either purposefully for embryo banking, if transfer was cancelled for overstimulation, or if no embryos were available for transfer). Donor cycles were included. More than one cycle per patient was included if applicable.

*Definitions* – Clinical pregnancy was defined by Society of Assisted Reproductive Technology (SART) criteria as the ultrasound presence of a fetal heart beat. Biochemical pregnancy was defined by the presence of a positive serum hCG, with or without an intrauterine gestational sac seen on transvaginal sonogram, but without fetal heartbeat. Implantation rate was defined as the number of fetal heartbeats per embryo transferred. Total pregnancy loss (SAB) was defined as the loss of all fetal heart beats previously identified. Pregnancy was defined by a positive hCG drawn 14 days after fresh egg retrieval or at the equivalent time frame after FET. Partial pregnancy loss (PSAB) was defined as a pregnancy with more than 1 sac seen on ultrasound (independent of the presence of cardiac activity) and a loss of one or more sacs but with the end result still being a live birth.

Data regarding patient characteristics (age, BMI, maximum FSH), IVF cycle parameters (estradiol levels, number of eggs retrieved, and endometrial thickness) and pregnancy outcomes (hCG level, number of sacs, clinical pregnancy, implantation rate, partial and total miscarriage rates, and live birth) were collected.

Statistical analysis was performed using Stata version 10 and a p-value <0.05 was considered statistically significant. *T*-test and Chi-square test were used to analyze patient and pregnancy data with linear and logistic regression when appropriate. The study was approved by the Institutional Review Board of Staten Island University Hospital.

## Results

Most patients were young (age 36.46 ± 5.21 years, n = 1130) with normal maximum FSH levels (8.26 ± 7.73 mIU/mL) and slightly elevated BMI (28.11 ± 7.40 (kg/m2). For all age groups clinical pregnancy rate was 37% and live birth rate was 30%. The overall prevalence of multiple pregnancies was 24% (n = 102) and the vast majority (n = 97) were twins. There were no differences in twin pregnancy rates (26%, n = 63 versus 21%, n = 34 p = 0.22) or high order multiples (1.6%, n = 4 versus 0.6%, n = 1, p = 0.37) between fresh and frozen cycles. Among fresh IVF cycles, 230 transfers took place at the blastocyst stage and 452 at the cleavage stage. Among FET cycles, 345 were blastocyst transfers and 103 were cleavage stage transfers. As expected, peak estradiol levels were higher with fresh cycles, and endometrial thickness was slightly higher as well (Table 1). Slightly more embryos were transferred in fresh cycles. Additionally, the initial hCG value was higher for FET cycles. Other patient characteristics did not differ between cycle types (Table 1). On average, patients froze 3.65 ± 4.86 embryos (n = 682) per fresh cycle.

**Table 1:** **Patient baseline characteristics by cycle type (fresh versus frozen)**

Characteristic	Fresh (n = 682)	Frozen (n = 448)	p-value
Endometrial Thickness(mm)* (mm)*	11.34 ± 2.96	10.62 ± 2.46	<0.01
Peak Estradiol (pg/mL)*	1872.58 ± 1274.64	424.03 ± 224.17	<0.01
Maximum FSH (mIU/mL)*	8.22 ± 6.42	8.33 ± 9.39	0.80
BMI(kg/m2)*	27.94 ± 7.40	28.38 ± 7.41	0.33
Age*	36.50 ± 4.96	36.40 ± 5.56	0.74
# Embryos Transferred*	2.53 ± 1.01	2.28 ± 1.01	<0.01
Positive Pregnancy Test (%)**	51.33% ± 5.00%	46.77% ± 5.00%	0.11
Initial hCG (mIU/mL)*	63.69% ± 110.73%	84.11% ± 148.14	<0.01
Clinical Pregnancy (%)**	37.59% ± 4.85%	36.83% ± 4.83%	0.80
Implantation Rate (%)**	24.29% ± 3.73%	23.42% ± 3.52%	0.69
SAB (%)**	6.30% ± 2.43%	6.92% ± 2.54%	0.68
Biochemical Pregnancy (%)**	9.82% ± 2.98%	14.29% ± 3.50%	0.02
Live Birth (%)**	30.65% ± 4.61%	30.13% ± 4.59%	0.86

*T-test for continuous data, **Chi squared for dichotomous data.

**Figure 1:**
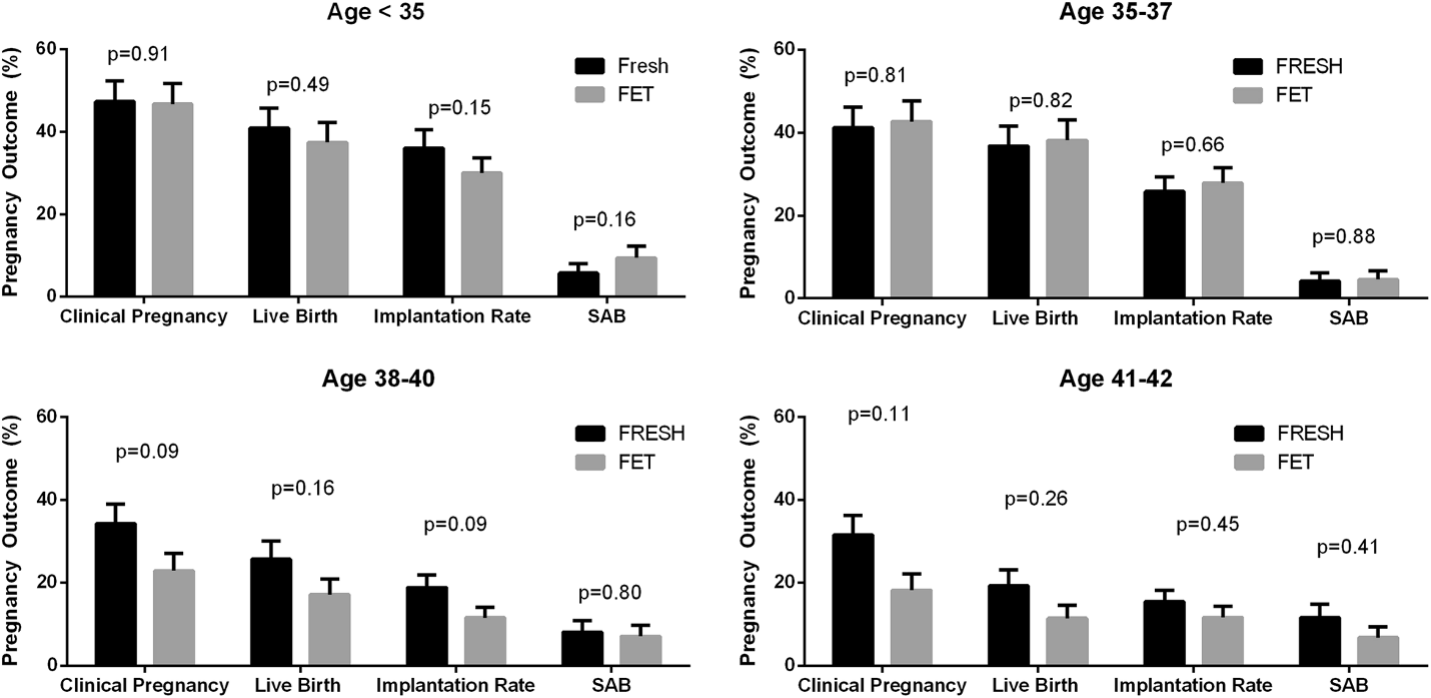
**Pregnancy outcomes by SART groups by cycle type.** Chi-Square test was used to analyze pregnancy outcome data by SART age categories. Clinical pregnancy, live birth rate, total pregnancy loss (SAB) and implantation rate were comparable in each age category between fresh and frozen cycles.

**Figure 2:**
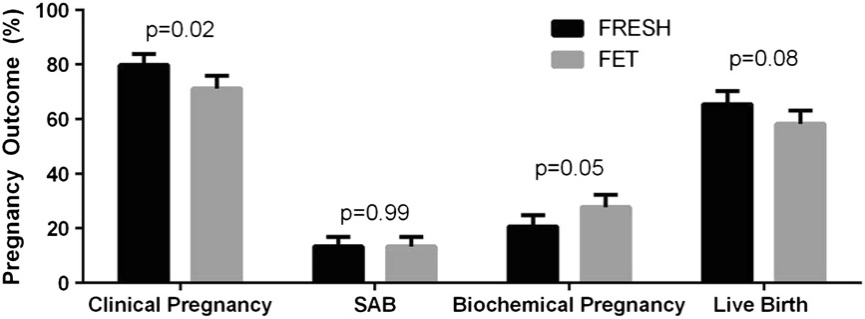
**Pregnancy outcome among patients with positive hCG by cycle type.** Chi-Square test was used to analyze pregnancy outcome data among patients who achieved a positive pregnancy test. Clinical pregnancy rate was slightly higher in fresh cycles.

**Figure 3:**
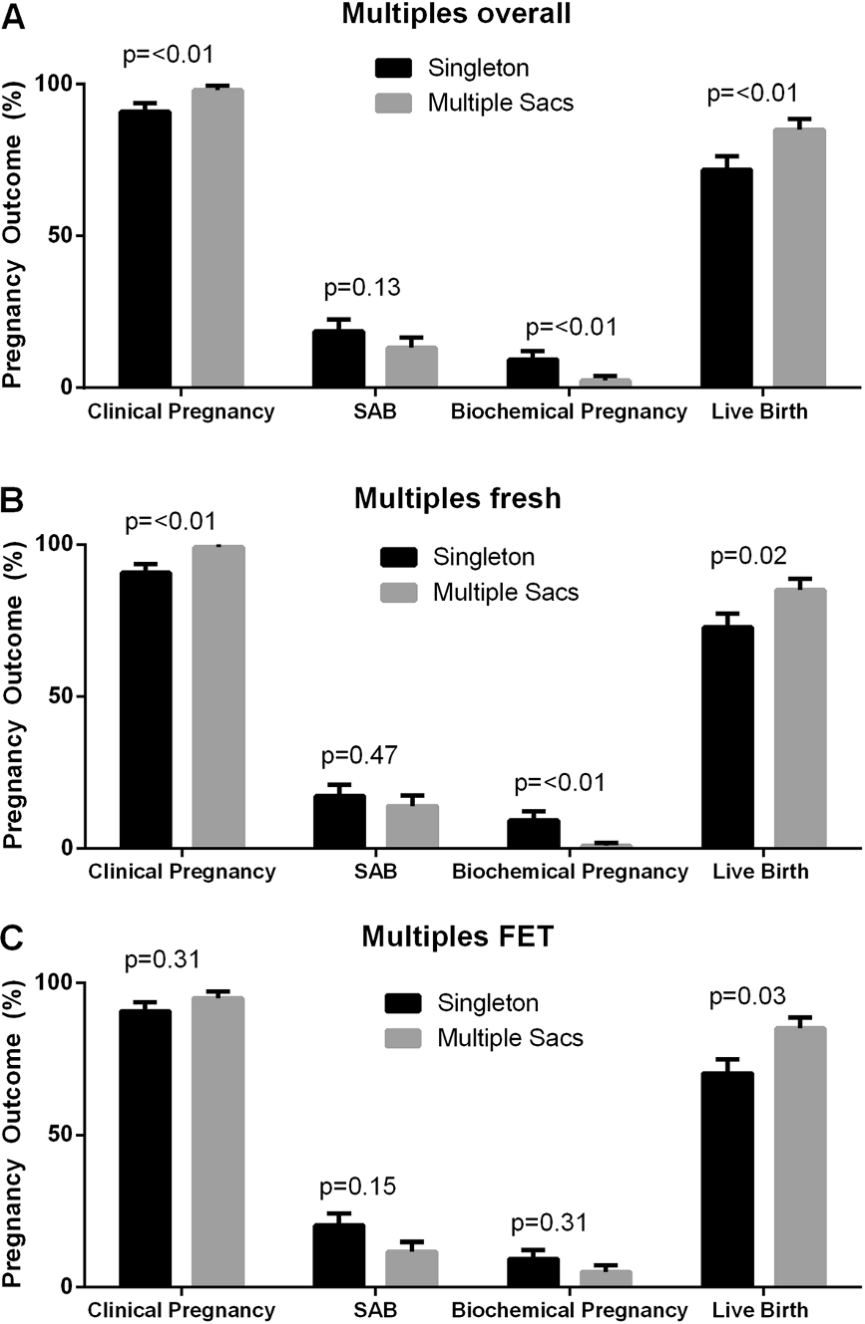
**Clinical outcome data in patients who achieved more than one sac on ultrasound.** Chi-Square test was used to analyze pregnancy outcomes for multiples pregnancies. Overall clinical pregnancy rate and live birth rates were higher in patients achieving multiple pregnancy sacs. These same relationships were seen in fresh cycles but only live birth rate was higher in frozen cycles.

Overall, pregnancy outcome data were similar between fresh and frozen cycles. Clinical pregnancy, implantation and live birth rates did not differ. Biochemical pregnancies were slightly more common for frozen cycles (Table 1). When analyzing pregnancy outcomes by SART reporting age groups, comparable outcomes were seen for clinical pregnancy, live birth, implantation rate, and SAB by cycle type (Figure 1). Overall, for every additional embryo transferred, the risk of multiple pregnancy rose (OR 1.31, p = 0.023). This was even more significant for patients under age 35 (OR 1.57, p = 0.019). Additionally, the chance of live birth was analyzed by number of embryos transferred (Table 2). For all patients, per given number of embryos transferred from 1 through 5, there was no difference in live birth rate between fresh and frozen cycles. This was also true for patients under age 35. For all patients, live birth rate was higher when 2 versus 1 embryos were transferred (OR 1.50, p = 0.048), but 3,4 and 5 embryos transferred compared to 1 did not increase live birth rate. Interestingly, when comparing 2 embryos versus 3,4 or 5 embryos transferred, more embryos decreased live birth rate (3, OR 0.67, p = 0.11; 4, OR 0.76, p = 0.012; 5, OR 0.61, p = 0.46). In patients under age 35, there was no relationship between live birth rate and number of embryos transferred (OR 0.96, p = 0.75).Among all patients with an initially positive hCG, approximately 76% achieved a clinical pregnancy and 62% achieved a live birth. Total pregnancy loss occurred in 14% and biochemical pregnancy rate was 24%. The initial hCG value was higher for FET cycles (136.63 versus 163.83, p = 0.03) and the number of sacs initially seen was slightly higher for fresh cycles (1.23 versus 1.07, p = 0.02). Although clinical pregnancy rates were higher for fresh cycles, live birth rates did not differ (Figure 2).Pregnancy outcomes differed between singleton and multiple pregnancies, defined by the presence of one or more intrauterine gestational sacs, independent of fetal cardiac activity. Clinical pregnancy and live birth rates were higher in pregnancies where more than 1 sac was initially identified, and biochemical pregnancy rate was significantly lower (Figure 3A). This held similarly true in fresh cycles (Figure 3B) but in frozen cycles (Figure 3C) only live birth rate was higher. There were a total of 168 patients who had more than 1 sac on initial ultrasound. Of those patients, 143 had a live birth (85%). However the rate of PSAB was 31% so a high proportion of patients with more than 1 sac delivered a singleton pregnancy. Of those patients with multiple sacs, only 54% eventually delivered multiples. Adjusting for age, FSH, and BMI, clinical pregnancy rate (OR 3.86, p = 0.02), live birth rate (OR 2.28, p < 0.01), SAB rate (OR 0.63, p = 0.10), and biochemical pregnancy rate (OR 0.26, p = 0.02) all favored having multiple sacs.

**Table 2:** **Live birth outcomes by number of embryos transferred**

Number of embryos transferred	Fresh cycles all ages (%)	Frozen cycles all ages (%)	p-value	Fresh cycles age <35 (%)	Frozen cycles age <35 (%)	p-value
1	26.7% (n = 75)	28.0% (n = 75)	0.86	40.5% (n = 37)	23.1% (n = 26)	0.15
2	37.4% (n = 302)	34.1% (n = 208)	0.45	47.0% (n = 134)	42.0% (n = 88)	0.47
3	27.9% (n = 197)	26.2% (n = 99)	0.76	24.4% (n = 41)	34.4% (n = 35)	0.34
4	20.6% (n = 87)	31.3% (n = 51)	0.16	31.3% (n = 16)	52.9% (n = 17)	0.21
5	7.1% (n = 14)	25.0% (n = 4)	0.32	N/A	N/A	

All data analyzed by chi squared and logistic regression.

However in patients under age 35 and in patients using donor egg (not shown), there were no differences in clinical pregnancy (OR 2.09, p = 0.28), live birth (OR 1.72, p = 0.16), SAB (OR 0.76, p = 0.53) or biochemical pregnancy (OR 0.48, p = 0.28) in patients with one or more sacs. This finding was true for both fresh and frozen cycles (all p > 0.05). For all patients, BMI increased the risk of SAB (OR 1.04, p = 0.048) and decreased live birth rate (OR 0.97, p = 0.064), without affecting clinical pregnancy rate (OR 1.00, p = 0.983). BMI had the most significant effect in patients under age 35 (SAB rate OR 1.13, p < 0.001; live birth rate OR 0.91, p = 0.001; clinical pregnancy rate OR 1.01, p = 0.822) and in patients age 35–37 (SAB rate OR 1.00, p = 0.001; live birth rate OR 0.94, p = 0.004; clinical pregnancy rate OR 1.00, p = 0.918).

## Discussion

Patients clearly wish to achieve the highest live birth rate per cycle. Physicians try to achieve this in the safest way possible. Most physicians, as well as SART reporting measurements, counsel patients on clinical outcome per cycle, per retrieval or per transfer. But patients often want to know what happens once they are pregnant. If they have a positive pregnancy test, what are the chances of live birth? If more than one sac is seen, what is the chance of miscarriage? And if multiple sacs are seen and one stops growing or loses a heartbeat, what are the chances that the other will lead to a live birth? This study helps patients answer those questions. Our study demonstrates higher live birth rates when more than one sac is initially seen in both IVF and FET cycles. However, this same advantage was not seen in the best prognosis patients, those under age 35 and those using donor egg. Additionally, in patients under age 35, transferring more than 1 embryo did not increase live birth rate. Prior studies have demonstrated excellent pregnancy rates in young women undergoing single embryo transfer [13, 14]. There was likely some bias in our data as those patients with the morphologically best embryos were more likely to undergo SET. Our study did not take into account embryo quality. However, given high pregnancy rates and high live birth rates after an initial positive hCG, these women should be further counseled that single embryo transfer provides high live birth rates once pregnant.

Our study confirmed previously limited literature showing a lower rate of total pregnancy loss for multiple gestations after IVF when compared with singleton pregnancies. We also show the same to be true now for FET cycles. When more than one sac was demonstrated on ultrasound, the ultimate outcome was more commonly a live birth. This occurred despite a relatively high loss rate of extra sacs seen in these early pregnancies.

Aneuploidy is the most likely explanation for these findings as well as for the high maintenance of live births rates in younger patients [15, 16]. Many studies advocate embryo quality is the pivotal factor for successful implantation after IVF [17, 18]. However, successful implantation does not necessarily mean maintenance of pregnancy. Our findings suggest that in older women, the chance of multiple sacs leading to the delivery of multiple babies is low and likely reflects aneuploidy. This suggests a more aggressive embryo transfer strategy for maximizing the actual chance of a live birth [19] in the absence of pre-implantation genetic screening.

We did not specifically examine prior history of miscarriages in our patients so it is possible that some patients had recurrent pregnancy loss. Some patients had more than one cycle included in our data series. Their pregnancy outcome data if predisposed to miscarriage could have negatively impacted FET data relative to other patients who used FET after achieving a live birth on their prior fresh IVF cycle.

## Conclusions

Despite maternal and fetal risks of multiple pregnancies, patients who achieve a positive pregnancy test with ART and have more than one pregnancy sac initially seen are more likely to deliver at least one baby. However, the absolute differences are small and are only seen in women over age 35. In patient under age 35, SET maximized live birth rate and minimized multiple pregnancies. So the best prognosis patients can be counseled that achieving implantation of a single embryo still maximizes live birth and minimizes maternal and fetal morbidly in both fresh and frozen cycles. Whether or not the difference in live birth rates in less favorable patients warrants the transfer of additional embryos is another dilemma.

## Abbreviations

ART: Artificial reproductive technology
BMI: Body mass index
FET: Frozen embryo transfer
IVF: In vitro fertilization
SART: Society of Assisted Reproductive Technology.
